# Short-Term Functional and Morphological Changes in the Primary Cultures of Trigeminal Ganglion Cells

**DOI:** 10.3390/cimb44030084

**Published:** 2022-03-08

**Authors:** Carla Pires Veríssimo, Lionete Gall Acosta Filha, Fábio Jorge Moreira da Silva, Harrison Westgarth, Juliana De Mattos Coelho Aguiar, Bruno Pontes, Vivaldo Moura-Neto, Parisa Gazerani, Marcos F. DosSantos

**Affiliations:** 1 Laboratório de Morfogênese Celular (LMC), Instituto de Ciências Biomédicas (ICB), Universidade Federal do Rio de Janeiro (UFRJ), Rio de Janeiro 21941-902, Brazil; verissimocp@gmail.com (C.P.V.); gallacosta@hotmail.com (L.G.A.F.); fabio.jorge.moreira@gmail.com (F.J.M.d.S.); jumcoelho@gmail.com (J.D.M.C.A.); brunoaccpontes@gmail.com (B.P.); vivaldomouraneto@gmail.com (V.M.-N.); 2 Laboratório de Biologia Tumoral (LBT), Instituto de Ciências Biomédicas (ICB), Universidade Federal do Rio de Janeiro (UFRJ), Rio de Janeiro 21941-902, Brazil; 3 Programa de Pós-Graduação em Neurociência Translacional, Instituto Nacional de Neurociência Translacional (INNT-UFRJ), Rio de Janeiro 20231-092, Brazil; 4 Laboratório de Virologia Molecular, Departamento de Genética, Universidade Federal do Rio de Janeiro (UFRJ), Rio de Janeiro 21941-902, Brazil; hwestgarth@gmail.com; 5 Programa de Pós-Graduação em Anatomia Patológica, Hospital Universitário Clementino Fraga Filho (HUCFF), Universidade Federal do Rio de Janeiro (UFRJ), Rio de Janeiro 21941-617, Brazil; 6 Laboratório de Biomedicina do Cérebro, Instituto Estadual do Cérebro Paulo Niemeyer (IECPN), Secretaria de Estado de Saúde, Rio de Janeiro 20231-092, Brazil; 7 Centro Nacional de Biologia Estrutural e Bioimagem (CENABIO), Universidade Federal do Rio de Janeiro (UFRJ), Rio de Janeiro 21941-902, Brazil; 8 Department of Life Sciences & Health, Faculty of Health Sciences, Oslo Metropolitan University, 0130 Oslo, Norway; parisaga@oslomet.no; 9 Department of Health Science & Technology, Faculty of Medicine, Aalborg University, 9220 Aalborg, Denmark; 10 Departamento de Prótese e Materiais Dentários, Faculdade de Odontologia, Universidade Federal do Rio de Janeiro (UFRJ), Rio de Janeiro 21941-617, Brazil; 11 Programa de Pós-Graduação em Odontologia (PPGO), Universidade Federal do Rio de Janeiro (UFRJ), Rio de Janeiro 21941-617, Brazil; 12 Faculdade de Odontologia, Universidade Federal do Rio de Janeiro (UFRJ), Cidade Universitária, Ilha do Fundão, Rio de Janeiro 21941-617, Brazil

**Keywords:** primary cell cultures, trigeminal ganglia, neurons, satellite glial cells, orofacial pain

## Abstract

Several studies have proved that glial cells, as well as neurons, play a role in pain pathophysiology. Most of these studies have focused on the contribution of central glial cells (e.g., microglia and astrocytes) to neuropathic pain. Likewise, some works have suggested that peripheral glial cells, particularly satellite glial cells (SGCs), and the crosstalk between these cells and the sensory neurons located in the peripheral ganglia, play a role in the phenomenon that leads to pain. Nonetheless, the study of SGCs may be challenging, as the validity of studying those cells in vitro is still controversial. In this study, a research protocol was developed to examine the potential use of primary mixed neuronal–glia cell cultures obtained from the trigeminal ganglion cells (TGCs) of neonate mice (P10–P12). Primary cultures were established and analyzed at 4 h, 24 h, and 48 h. To this purpose, phase contrast microscopy, immunocytochemistry with antibodies against anti-βIII-tubulin and Sk3, scanning electron microscopy, and time-lapse photography were used. The results indicated the presence of morphological changes in the cultured SGCs obtained from the TGCs. The SGCs exhibited a close relationship with neurons. They presented a round shape in the first 4 h, and a more fusiform shape at 24 h and 48 h of culture. On the other hand, neurons changed from a round shape to a more ramified shape from 4 h to 48 h. Intriguingly, the expression of SK3, a marker of the SGCs, was high in all samples at 4 h, with some cells double-staining for SK3 and βIII-tubulin. The expression of SK3 decreased at 24 h and increased again at 48 h in vitro. These results confirm the high plasticity that the SGCs may acquire in vitro. In this scenario, the authors hypothesize that, at 4 h, a group of the analyzed cells remained undifferentiated and, therefore, were double-stained for SK3 and βIII-tubulin. After 24 h, these cells started to differentiate into SCGs, which was clearer at 48 h in the culture. Mixed neuronal–glial TGC cultures might be implemented as a platform to study the plasticity and crosstalk between primary sensory neurons and SGCs, as well as its implications in the development of chronic orofacial pain.

## 1. Introduction

Orofacial pain represents a highly generic term, since it refers to a large and heterogeneous group of painful syndromes that affect the face and oral cavity, as well as the related structures. In fact, some authors report that a considerable part of the general population will experience at least one episode of orofacial pain during their lifetime [1,2]. Nonetheless, the pathogenesis of orofacial pain has not been completely elucidated. Therefore, there is no consensus regarding the treatment of each subtype of orofacial pain disorder [3,4,5]. In addition, the coexistence of psychiatric diseases, and the occurrence of peripheral and central sensitization, make the treatment of orofacial pain extremely challenging in clinical practice, and make proper study of their complex mechanisms extremely difficult [6,7]. The multifaceted pathophysiological mechanisms involved in the pathogenesis of each subtype of orofacial pain have not been yet elucidated. In addition, the coexistence of psychological factors, along with the vast number of peripheral and central mechanisms, make the treatment of orofacial pain, and especially chronic orofacial pain, extremely challenging [6,7].

There is scientific evidence that the pathophysiology of orofacial pain not only involves the neuroplastic changes that affect the central nervous system (CNS), but also changes that affect the functioning of the peripheral nervous system (PNS) [8]. In fact, several studies have researched the role of peripheral neurons, satellite glial cells (SGCs), and the crosstalk between those cells inside the dorsal root ganglia (DRG), as well as within the trigeminal ganglia (TG), regarding the mechanisms of both nociceptive and neuropathic pain [9,10,11,12,13,14]. Therefore, studying the structure and function of the major components present in the DRG and TG is paramount to understanding the pathophysiology associated with the different painful syndromes that induce orofacial pain. The somatosensory nervous system comprises all parts of the nervous system involved in the processing and detection of different sensory stimuli modalities. With regards to pain, the noxious stimuli are detected by nociceptors, which are, in turn, spread throughout the PNS. The cell bodies of primary afferent neurons within the peripheral sensory ganglia (e.g., the DRG and TG) are surrounded by a specific type of peripheral glial cells, namely SCGs. SCGs arise from neural crest derivative boundary cap cells. Although it has been proven that SGCs genetically resemble Schwann cells, their functional roles in the PNS are similar to those of astrocytes at the CNS level [15,16]. The similarities between SGCs and astrocytes include the expression of glial markers, such as glutamine synthase, P2X7, and the glial fibrillary acid protein (GFAP). Those aspects are partially found in other neural crest-derived glial cells, such as the enteric glia [17]. However, the GFAP is only expressed in SGCs in cases of peripheral nerve injury. SGCs play an important role in the removal of extracellular K+, ensuring that sensory neurons do not become hyperexcitable [15]. Furthermore, SGCs take up glutamate from the extracellular space, mainly through the glutamate transporter, GLAST. Through the enzyme glutamine synthase, SGCs convert glutamate into glutamine and release it into the extracellular space. In the extracellular space, the neurons can take up glutamine and convert it into glutamate for their own use [18]. Despite these similarities, the SGCs present a fundamental difference from astrocytes, which is the formation of an intimate envelope surrounding the cellular bodies of sensory ganglia neurons, forming a unique neuron–glia unit [15,19]. The space between SGCs and the neuronal surface in the peripheral ganglia is nearly 20 nm. This is very close to that of the synaptic cleft. This organization is crucial to understanding neuron–SGC interactions [15,20,21].

Primary afferent neurons transmit sensory information from the periphery to the CNS, where both pain-processing and perception take place [15,22,23]. More specifically, the TG is responsible for the sensory innervation of most parts of the head, including the face, teeth, and tongue, among other structures. The territory of the trigeminal nerve can be divided according to its main branches or divisions, e.g., ophthalmic (V1), maxillary (V2), and mandibular (V3) [24,25]. Each division ramifies into several branches that provide the innervation of the majority of the face and head structures. The chronic constriction of the infraorbital nerve, the terminal branch of V2, and other similar surgical approaches have been largely used as experimental models of trigeminal neuropathic pain [26,27]. Interestingly, a recent study that induced trigeminal neuropathic pain in rats through a partial transection of the infraorbital nerve reported a decreased pain threshold, together with a lower expression of the small conductance calcium-activated potassium channel 3 (KCNN3 or Sk3) in the TG of the animals that underwent the infraorbital ligation (the experimental group) when compared to the control (sham) group [26,27]. In addition, the facial pain threshold of the experimental group was altered by the administration of the SK3 channel agonist, CyPPA, and the administration of the SK3 channel antagonist, apamin [27]. These findings suggest that SK3 might play an important role in trigeminal neuropathic pain and could be a potential target in future treatments for pain of a neuropathic origin. Remarkably, SK channels are widely expressed in the PNS and CNS, as well as in other tissues [28,29]. Nonetheless, the specific functions of each SK channel in the neural transmission, or in the nociception, have not been clarified [30]. The specific cell location of SK3 in the peripheral ganglia is still a matter of debate in the literature. For example, SK3 immunoreactivity is described in peripheral neurons [30,31]. Conversely, according to the results of other studies, SK3 is confined to the SGCs [31,32].

Based on these concepts, the primary cell cultures of sensory ganglia provide an experimental platform wherein the morphology, physiology, and, importantly, the cell-to-cell interactions between primary sensory neurons and SGCs can be explored in a well-controlled environment [33]. Although several studies have been conducted on cultured neurons and satellite glial cells (SGCs) [34,35], the validity of the use of primary sensory ganglia cultures to study the interactions between neurons and SGCs has been a matter of debate in the literature [35]. This occurs, at least in part, due to the fact that, after a few hours in culture, phenotypic changes might take place in the SGCs [35]. Those modifications include morphological changes in the expression profile of some of the proteins found in such types of cells, when compared to the characteristics of the same cells found in vivo. For example, after several hours in culture, an in vitro decrease was found in the expression of GS, the main immunomarker of SGCs in vivo, along with important morphological changes, such as the development of the spindle-shaped format [35]. Furthermore, the migration of those cells away from their original location, modifying their intimate anatomical relationship with the cell bodies of sensory neurons, has been previously reported. This feature is probably the most remarkable change that takes place in cultured TGCs [35]. Recent studies have also applied novel approaches to study the role of SGCs in health and disease, especially in neuropathic pain [36,37,38]. Nevertheless, many open-ended questions remain in this field. For example, the expression of purinergic receptors, which are important in the crosstalk between neurons and SGCs, was demonstrated in the TG [39]. However, the expressions of other important receptors in the neurons and SGCs of the peripheral ganglia have scarcely been explored. In this regard, changes in the expression of Sk3 over time has never been explored in these cells in vitro. Furthermore, the characterization of the morphological changes that occur in SGCs in vitro has never been studied by scanning electron microscopy (SEM). These features were explored and constitute the main focus of the present study.

## 2. Materials and Methods

### 2.1. Animals

All experimental procedures were approved by the Committee on Ethics in the Use of Animals of the Federal University of Rio de Janeiro (UFRJ), Brazil (approval no. DAHEICB096-10/16). A total of seven neonate (P10–P12) Swiss male mice were used in each in vitro experiment. In addition, one animal was used for anatomical and conventional histology characterization. The animals were euthanized with an overdose of isoflurane. The isoflurane vaporizer was adjusted to a 5% flow rate. Euthanasia was confirmed with cervical dislocation, followed by decapitation. This study strictly followed the ARRIVE guidelines for reporting animal research [40].

### 2.2. TG Dissection

Before decapitation took place, each animal was disinfected with alcohol 70% (*w*/*v*). Upon the completion of this process, the skin and the subcutaneous tissue surrounding the head of each animal was carefully removed and the skull was transferred into a 90-mm Petri dish containing ice-cold phosphate-buffered saline solution (PBS).

**Figure 1:**
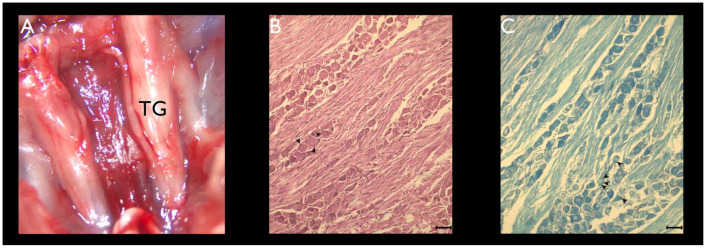
Dissection of the TG and histological characterization of TGCs. (**A**) Macroscopic aspect of the right and left TGs after the dissection of the skullcap, the skin (along with the subcutaneous tissue), and the brain (superior view). (**B**) Optical microscopy of a TG section stained with H&E, captured under 40× magnification. (**C**) Optical microscopy of a TG section stained with LFB, captured under 40× magnification. Arrowheads (**B**,**C**) represent SGCs. Scale bars (**B**,**C**) represent 100 µm.

Each Petri dish was then transferred to a laminar flow cabinet and the mice skulls were cut open to expose the brain, which was folded back to visualize the skull base and to individualize the TG. Each TG (two per animal) was then dissected using ophthalmic forceps, under a stereoscopic microscope (Leica *EZ4* W, Leica Microsystems, Wetzlar, Germany) (Figure 1A). Each TG was also cleaned from the surrounding connective tissue and blood vessels.

### 2.3. Primary Culture of TG Cells

Each dissected TG was placed into a 35-mm Petri dish containing 1 mL of DMEM/F12 (Dulbecco’s Modified Eagle’s Medium, Gibco, Thermo Fisher Scientific, Inc., Waltham, MA, USA). This procedure was followed by enzymatic digestion with 250 µL of Trypsin (Gibco, Thermo Fisher Scientific, Inc., Waltham, MA, USA), 50 µL of Collagenase Type II (Gibco, Thermo Fisher Scientific, Inc., Waltham, MA, USA), and 20 µL DNAse (DN25, Sigma-Aldrich, St. Louis, MO, USA) and kept in a humidified 5% CO_2_ incubator at 37 °C for 20 min. After this process, the mechanical dissociation was performed using Pasteur and a P1000 pipette. The purpose of this step was to obtain a homogeneous solution. Then, this resulting cell suspension was centrifuged and resuspended in 10 mL of DMEM/F12, supplemented with glucose (33 mM), glutamine (2 mM), sodium bicarbonate (3 mM), penicillin/streptomycin (0.5 mg/mL), fungizone (2.5 μg/mL), and 10% fetal bovine serum.

The final suspension containing the TGCs was then distributed onto poly-L-lysine (Sigma-Aldrich, St. Louis, MO, USA)-treated glass coverslips in a 24-well plate. This plate was kept in optimal culture conditions (37 °C and in a humidified atmosphere with 5% CO_2_ level) for 48 h.

### 2.4. Histochemistry

The TG sections of 15–20 μm were obtained to permit a full histological characterization of the neonate mice’s trigeminal ganglion. The TG samples were first fixed with 4% formaldehyde for 16 h at 4 °C. After that, they were washed with PBS and incubated in 30% sucrose (diluted in PBS) for at least 16 h. The samples were then transferred to an optimal cutting temperature (OCT) compound (Tissue-Tek, Sakura Finetek, Torrance, CA, USA) mold, and were promptly frozen by immersion in liquid nitrogen. A cryostat (Leica CM1860, Leica Microsystems, Wetzlar, Germany) was set to a temperature of −20 °C, and 15–20-μm sections of the frozen blocks were obtained. The sections were transferred to histology slides that were previously treated with poly-L-lysine (Sigma-Aldrich, St. Louis, MO, USA).

A standard protocol for Hematoxylin–Eosin (H&E) staining was applied. This includes dewaxing, followed by hydration; hematoxylin staining for four minutes; differentiation with 1% acid alcohol; bluing in saturated lithium carbonate solution; counterstaining with 1% eosin for 30 s; dehydration; clearing; and cover-slipping. In addition, Luxol Fast Blue (LFB) staining was applied in the TG sections. A slightly different protocol was performed for this, which included dewaxing, hydration, and staining with LFB at 58 °C overnight, as well as differentiation, using lithium carbonate solution for three minutes, and continuing the differentiation with 70% alcohol solution, until the grey matter and the white matter could be clearly distinguished. Finally, for cresyl violet, the TG sections were submerged into the staining solution for 5 to 10 min.

### 2.5. Phase-Contrast Microscopy

The short-term morphological changes that occurred in cultured TGCs (e.g., changes found within a period of 48 h) were evaluated with phase-contrast microscopy, using an inverted microscope (Leica DMI-4000B, Leica microsystems, Wetzlar, Germany). The TGC cultures were photographed at three timepoints: 4 h, 24, and 48 h.

### 2.6. SEM

For SEM, sample processing was performed using 13-mm diameter round glass coverslips, pre-coated with poly-L-lysine (Sigma-Aldrich, St. Louis, MO, USA), as previously described. Each coverslip containing the TGCs was fixed for 1 h in 2.5% glutaraldehyde, diluted in cacodylate buffer (0.1 M (pH 7.2)), washed three times with cacodylate buffer 0.1 M, and then post-fixed in the dark for 1 h with 2% osmium tetroxide (OsO_4_) diluted in a cacodylate buffer 0.1 M, washed three times with cacodylate buffer 0.1 M, and dehydrated in a graded alcohol series (30%, 50%, 70%, 90%, and three exchanges of 100% ethanol, with each step taking a total of 15 min). The samples were critical-point dried (CPD 030 Bal-tec, Balzers, Liechtenstein), mounted on specific supports, and metalized with gold using a specific metallizer (EM SCD 050, Leica microsystems, Wetzlar, Germany) with a thickness of 18 nm. The visualization was further performed in a Zeiss EVO 40 VP scanning electron microscope [32].

### 2.7. Time-Lapse Photography and Analysis

The TGCs were plated on culture dishes and were maintained in a culture chamber with controlled temperature conditions and a controlled CO_2_ concentration (37 °C and 5%, respectively), and were adapted to an inverted microscope, Leica DMi1 (Leica microsystems). During the 48 h, phase-contrast images were captured every 10 min using a HD camera (Leica MC170, Leica microsystems, Wetzlar, Germany). After the movie assembly, 20 different individual cells were marked with black dots using the Image J software (National Institutes of Health, Bethesda, MD, USA). Then, their labeled trajectories were obtained, and their migration velocities were determined.

### 2.8. Immunocytochemistry

Coverslips from each of the three analyzed timepoints, 4 h, 24 h, and 48 h, were washed twice with ice-cold PBS and were fixed in 100% ice-cold methanol (−20 °C) for 5 min at room temperature. Following this process, each coverslip was washed in ice-cold PBS for 5 min.

For immunocytochemistry with the conjugated antibody anti-SK3-ATTO-594 (Alomone, Jerusalem, Israel), a blocking buffer solution (PBS, 10% goat serum and 0.05% Triton X-100) was prepared. Each coverslip was then immersed in this solution for one hour. After that, the anti-SK3 antibody was added to the blocking buffer at a 1:100 dilution and was incubated overnight at 4 °C [36].

For immunocytochemistry with the primary antibody rabbit anti-βIII-tubulin (Abcam, Cambridge, UK), the coverslips were first permeabilized with 0.2% Triton X-100 for 5 min at room temperature, and they were then blocked with 5% bovine serum albumin (Sigma-Aldrich, St. Louis, MO, USA) for 1 h. The rabbit anti-βIII-tubulin antibody was then added to the blocking buffer at a 1:100 dilution and was incubated overnight at 4 °C. After incubation with the primary antibody, the coverslips were washed with PBS and were then incubated with the secondary antibody, the goat anti-rabbit Alexa Fluor 488 (1:400) (Molecular Probes ™, Eugene, OR, USA), at room temperature for 1 h [37].

Nuclei were counterstained with DAPI (4′,6-diamidino-2-phenyindole, dilactate, Sigma-Aldrich, St. Louis, MO, USA). The images were obtained with a Leica DMi8 inverted fluorescent microscope. The deconvolution of the images and the Z projection analysis was performed. Each of those images was obtained with the projection of at least five photos along the *Z*-axis. [37].

The fluorescence quantifications for SK3 were performed using ImageJ. In brief, outlines were drawn around each cell, and the values for the area $\left(A_{C}\right)$ and the mean fluorescence value and integrated density (ID) were obtained, along with the corresponding measurements for the adjacent background $\left(F_{B}\right)$. Then, the corrected total cell fluorescence (CTCF) was determined for each cell following the equation: $CTCF=ID-\left(A_{C}\times F_{B}\right)$. The mean CTCF values were plotted for each timepoint.

### 2.9. Statistical Analysis

A box-and-whisker plot was used for the migration velocity representation. The box extended from the 25th to 75th percentiles, with a black horizontal line at the median; black whiskers extended from the 5th to the 95th percentiles.

A bar graph (mean ± standard error) was used to represent the SK3 fluorescence quantification timepoints. Data were analyzed using the GraphPad Prism statistics software 8.1 (GraphPad Software, Inc., La Jolla, CA, USA). A one-way ANOVA, followed by Tukey’s test, was used for comparisons between each situation, where * means *p* < 0.05 and **** means *p* < 0.0001. The *p*-values and other numbers are provided in the figure legends.

## 3. Results

The results of the histochemistry and immunocytochemistry are presented separately. The results of the phase-contrast microscopy, SEM, and time-lapse photography analyses are presented together in order to provide a better understanding of the findings.

### 3.1. Histochemistry

An optical microscopy of the TG sections revealed the presence of a parenchymal tissue essentially composed of large-, medium-, and small-cell bodies of pseudounipolar neurons. It was also possible to distinguish groups of 4–5 flattened cells surrounding each cell body of a pseudounipolar neuron that was embedded in the connective tissue of the TG. These are the SGCs (Figure 1B,C). Remarkably, the cell bodies of pseudounipolar neurons were predominantly clustered in the outer (cortical) region of the TG. Conversely, the inner (medullary) region of the TG was mostly occupied by nerve fiber bundles.

### 3.2. Phase-Contrast Microscopy, SEM, and Time-Lapse Photography Analyses

**Figure 2:**
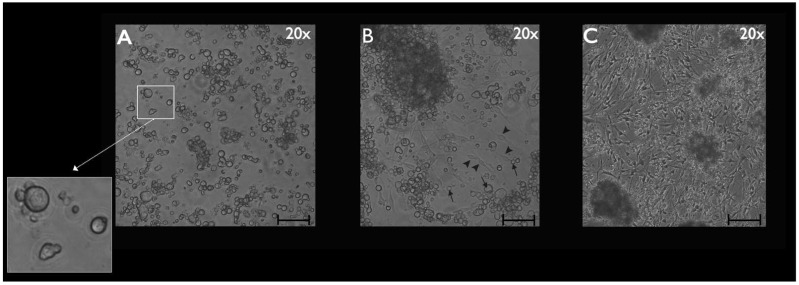
Phase-contrast microscopy of primary cultures of TG after 4 h (**A**), 24 h (**B**), and 48 h (**C**). Within the first four hours, a close relationship between the SGCs and the cell bodies of neurons was detected. This relationship was highlighted in the white square on the left side of Figure 1A (a 3.0 zoom was adopted for better visualization). After 24 h, the majority of the cultured TG SGCs adopted a fusiform shape. (**B**): arrows indicate neurons, arrowheads indicate SGCs. After 48 h, they migrated away from the neurons to which they were originally related and arranged themselves in parallel bundles. Scale bars represent 50 µm.

**Figure 3:**
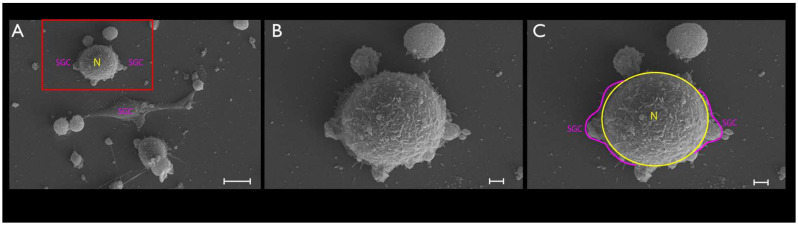
The same pattern found with phase-contrast microscopy was demonstrated with the SEM of the TG cultures (**A**–**C**). To obtain a better visualization, SGCs were highlighted in pink, and the neurons in yellow. SCGs lost their proximity to the neurons at this stage. Scale bars represent 10 µm in (**A**) and 2 µm in (**B**,**C**).

**Figure 4:**
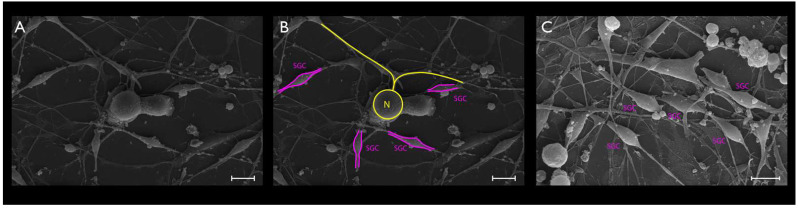
The TG cultured cells after a period of 24 h (**A**–**C**). Pseudounipolar neurons could be clearly visualized (yellow, **B**). At this stage, SGCs clearly adopted a fusiform shape and were located at short distances from the TG neurons (pink, **B**). Scale bars represent 10 µm.

**Figure 5:**
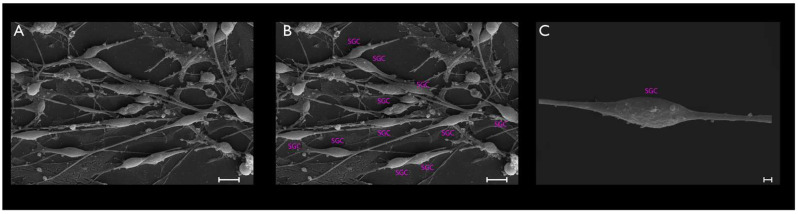
The TG cultured cells after 48 h (**A**–**C**). Pseudounipolar neurons exhibited elongated and thin processes. SGCs migrated away from those neurons, maintaining a fusiform shape. Furthermore, some of those cells were arranged in parallel bundles. Scale bars represent 10 µm in (**A**,**B**) and 1 µm in (**C**).

**Figure 6:**
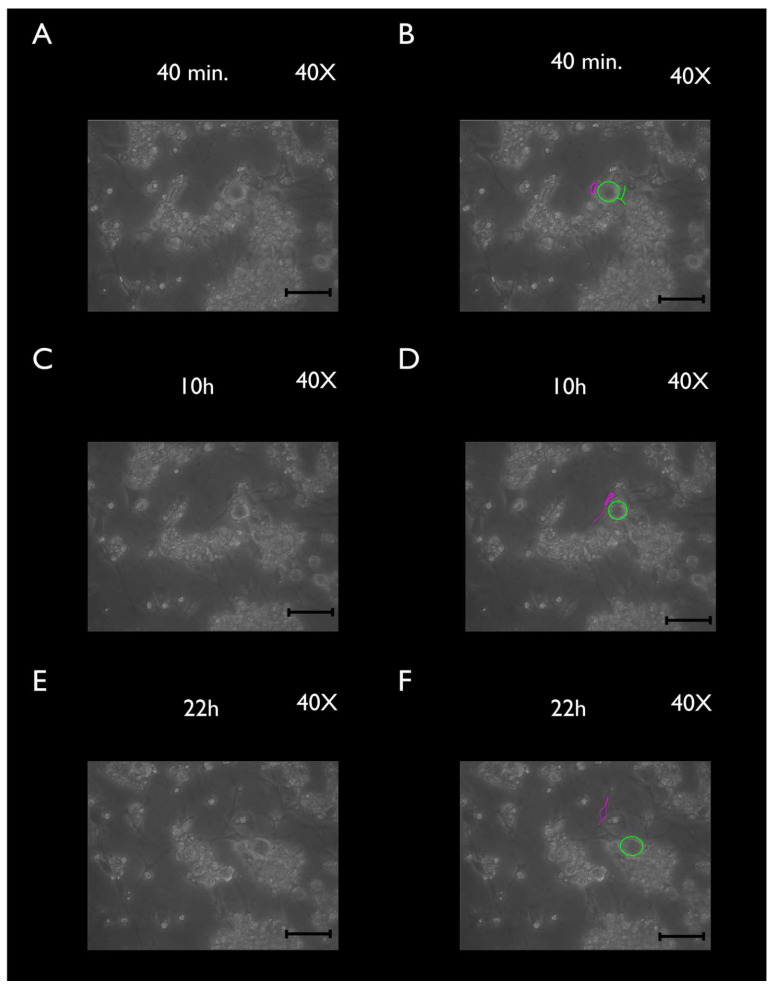
Time-lapse frames of cultured TGCs during the first 22 h (40× magnification). Pseudounipolar neurons (green) exhibit a round shape. At 40 min (**A**,**B**), SGCs (pink) maintain their proximity to neurons. However, at 10 and 22 h (**C**–**F**) they start migrating away from those neurons. In addition, they start developing a fusiform shape. Scale bars represent 100 µm.

**Figure 7:**
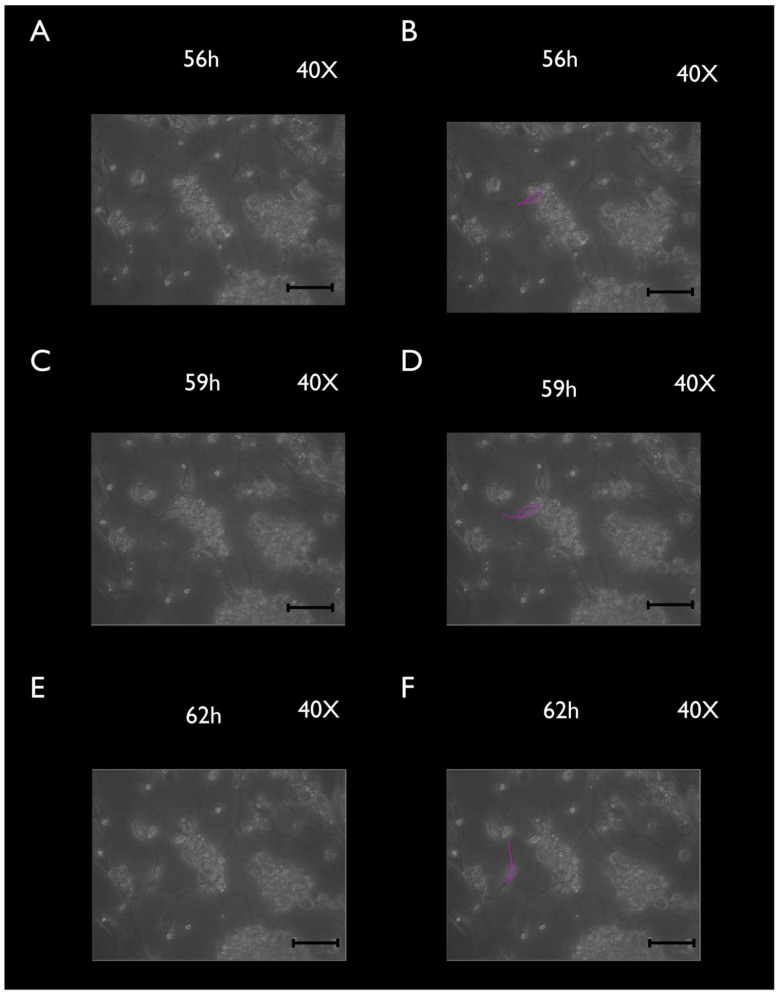
Time-lapse frames of cultured TGCs at 56, 59, and 62 h (40× magnification). SGCs continue to migrate away from neurons and develop a clear fusiform aspect (**A**,**C**,**E**). SGCs are highlighted (pink, **B**,**D**,**F**). Scale bars represent 100 µm.

**Figure 8:**
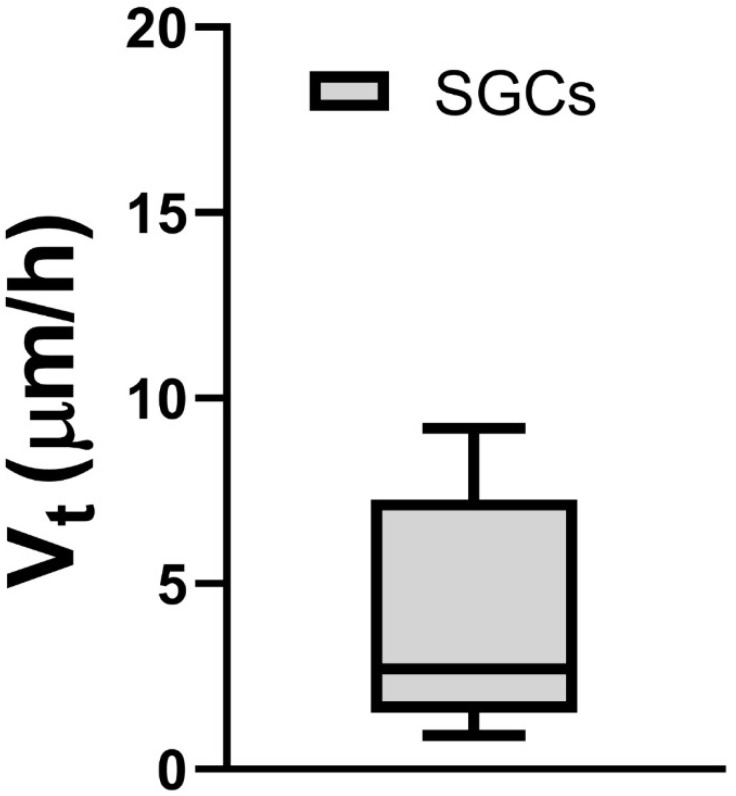
Migration velocity of SGCs.

Cultured TGCs were then evaluated at the following timepoints: 4, 24, and 48 h. Upon phase-contrast microscopy and SEM evaluation, the following short-term morphological changes were found in cultured TGCs: within the first 4 h, each TG neuron exhibited a round shape with a relatively large size, whereas the SCGs were clearly smaller, were also round-shaped, and were in close contact with the analyzed TG neurons (Figure 2A and Figure 3A–C). After 24 h, cultured TG neurons developed long and thin processes, while each SGC showed a flattened and fusiform shape. At this point, the SGCs were found at short distances from the neurons (Figure 2B and Figure 4A–C). After 48 h, neurons kept their elongated and thin processes, while the SGCs migrated away from those neurons. At this stage, the SGCs maintained a fusiform shape. In addition, some of those cells were arranged in parallel bundles (Figure 2C and Figure 5A–C). Similar changes were confirmed by time-lapse photography (Figure 6 and Figure 7). In addition, a videomicroscopy analysis allowed for a quantitative determination of the migration velocity of the SGCs (4.23 ± 0.72 µm/h) (Figure 8).

### 3.3. Immunocytochemistry Evaluation

**Figure 9:**
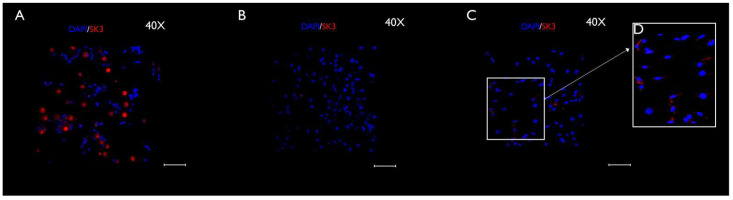
Anti-SK3 immunocytochemistry carried out in cultured TGCs (40× magnification), after 4 h (**A**), 24 h (**B**), and 48 h (**C**). SK3 was represented in red, while the nuclei (immunostained with DAPI) were depicted in blue. The expression of SK3 changed over time. Hence, it was more intense in the early 4 h, decreasing with 24 h, and rising again around 48 h. At this last point, SK3 was presumably concentrated in SGCs. The white square (**D**) shows a cluster of SGCs (a 1.5 zoom was adopted for better visualization). Scale bars represent 50 µm. CTCF = corrected total cell fluorescence.

**Figure 10:**
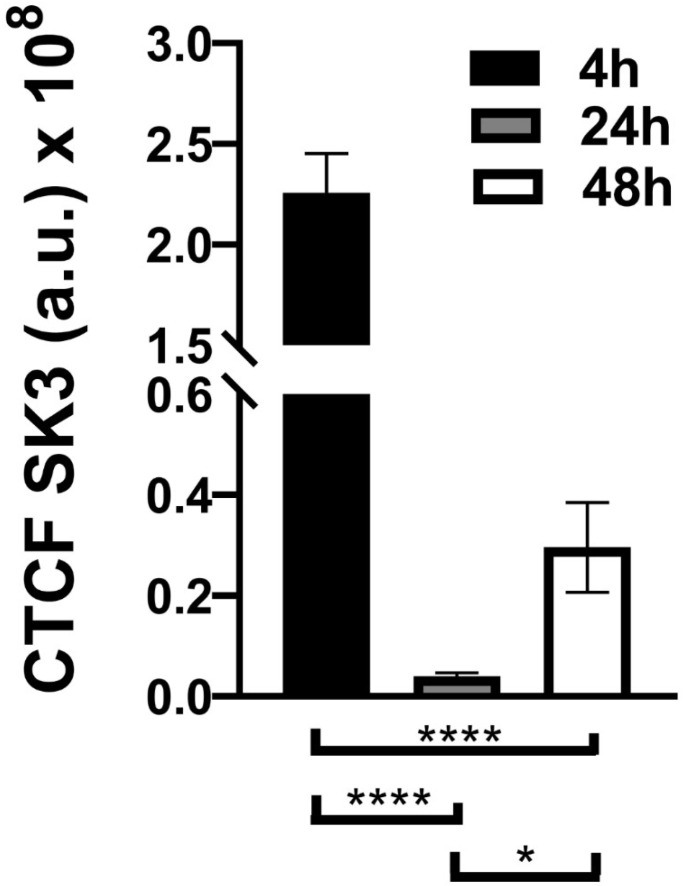
SK3 fluorescence quantification at each time point (4 h, 24 h, and 48 h). * *p* < 0.05, **** *p* < 0.0001.

**Figure 11:**
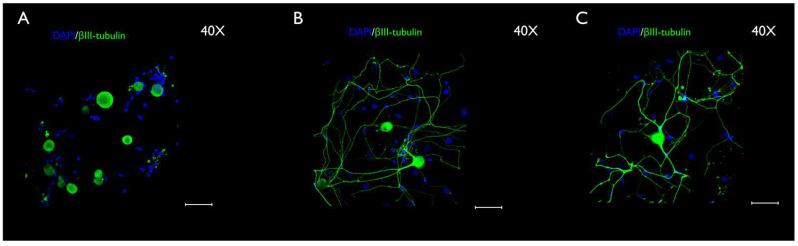
Anti-βIII-tubulin immunocytochemistry performed in cultured TGCs (40× magnification), after 4 h (**A**), 24 h (**B**), and 48 h (**C**). A typical pattern of peripheral neuron immunostaining is illustrated in green. Nuclei immunostaining with DAPI is represented in blue. Over the interval analyzed, neuronal changes, from a round shape, to a highly ramified shape, were observed. Scale bars represent 50 µm.

The neurons and SGCs were differentiated by immunocytochemistry. The SGCs were identified by immunostaining for SK3, which has been used as a specific marker of SGCs in vivo, and βIII-tubulin, a typical neuronal marker, both in vivo and in vitro. Moreover, DAPI staining contributed to differentiating the neurons and SGCs by nuclei sizes. For instance, neurons showed large and faintly stained nuclei, whereas SCGs exhibited small and bright nuclei. Immunocytochemistry revealed changes in SK3 staining over time (Figure 9A–C). The SK3 immunostaining was more intense at the early evaluation (4 h), decreasing at the subsequent stage (24 h), and then rising again during the last period of evaluation (48 h) (Figure 9A–C). A statistically significant difference was found in the quantification of the immunostaining during the study period (4 h vs. 24 h), as follows: 4 h and 24 h (*p* < 0.0001), 4 h and 48 h (*p* < 0.0001), and 24 h and 48 h (*p* < 0.05). Figure 10 represents the SK3 fluorescence quantification. Figure 11 illustrates βIII-tubulin staining at 4 h (Figure 11A), 24 h (Figure 11B), and 48 h (Figure 11C). In addition, a double-staining was performed with SK3 and βIII-tubulin. The results showed a group of cells that were positive for both SK3 and βIII-tubulin. However, some cells were only stained with βIII-tubulin, while others were only stained with SK3. This pattern was found in all stages and is presented in the Supplementary Materials (Figure S1A–C).

Phase-contrast microscopy allowed the identification of short-term morphological changes (at three 4 h, 24, and 48 h) that take place in the TG cells in vitro (Figure 2A–C). The coverslips used to perform the phase-contrast microscopy analysis were fixed, and the immunocytochemistry was conducted at each timepoint (Figure 9A–D, Figure 11A–C and Figure S1A–C). The immunocytochemistry permitted the identification of changes that occur in the immunostaining of the specific markers, Sk3 and βIII-tubulin, at the three timepoints: 4 h, 24, and 48 h.

## 4. Discussion

The etiology and physiopathology of painful syndromes are complex and diverse. For many years, SGCs received little attention, despite the presence of sensory ganglia studies [25]. However, recent works suggest a role of peripheral glial cells, particularly SGCs, and the crosstalk between such cells and sensory neurons located in the peripheral ganglia, in the whole phenomenon, which leads to pain development and chronification [41]. In fact, the intercellular commination between SGCs and neurons, especially through GAP junctions and the purinergic receptors P2Rs, seems to play a major role in the mechanisms of both inflammatory and neuropathic pain. A model was proposed to explain the mechanisms by which increased gap junctional communication and the sensitization to ATP could be part of the role that SGCs exert in the neuronal hyperexcitability found in different experimental pain models [20]. Not surprisingly, SGCs have been considered potential targets in the development of novel pain therapies [42].

The space between SGCs and the neuronal surface in the peripheral ganglia is nearly 20 nm. This is very close to that of the synaptic cleft. This organization is crucial to understanding the neuron–SGC interactions [15,20,21]. However, the molecular study of SGCs in vivo is a difficult task, due to this close physical relation to the primary sensory neurons and other cellular components of the sensory ganglia.

To circumvent this challenge, primary SGC cultures were established [22,43]. Nonetheless, the validity of the in vitro studies of SGCs remains controversial, since some authors have verified that SGCs change their phenotype, gene expression, and function in vitro [25,43], complicating the proper interpretation of the obtained results.

It has been reported that SGCs, as well as other neural crest-derived cells, exhibit enormous plasticity in vitro [31,44]. The fact that SCGs derive from neural crest cells (more specifically, boundary cap cells) could explain their possible role as progenitor cells. Notably, it has been shown that cultured embryonic DRG SGCs may develop into either Schwann cells or into oligodendrocytes. This could probably be explained by the fact that, when removed from their regular environment, SGCs lose their interactions with the major components of the sensory ganglia and, more importantly, they lose their intimate contact with the cell bodies of peripheral sensory neurons [45]. Interestingly, other studies have also suggested that the origin of the cell progenitors that give rise to both neurons and glial cells in the peripheral ganglia may link to SGCs. In the current study, SGCs expressed similar progenitor markers and molecular features to the radial glia and the neurogenic glial precursors found in the adult CNS [46].

Nevertheless, some of the findings reported by the works that studied the changes that occur in the peripheral ganglia cells in vitro are very likely region- and species-dependent. Furthermore, it has been hypothesized that such results significantly vary according to the age of the animals providing cells for such studies [12]. Finally, even small technical differences might affect the obtained findings. For example, glutamine synthetase (GS) is a widely used SGC marker both in vivo and in vitro studies [22,33,47]. Nonetheless, the possible variability in the expression of GS in cultured peripheral ganglia cells, over time, is still a controversial issue. For instance, according to some authors, there is no evidence that important changes occur in the expression of GS within a period of 21 days of primary SGC cultures isolated from mice TGs [33]. On the other hand, other authors have found that the expression of GS rapidly declines in SGCs within the first 48 h in culture [35]. Therefore, it is still necessary to define and interpret the possible in vitro changes that occur in different SGCs markers that are usually used in vivo.

Although the immunoreactivity of SK3 has been demonstrated in both peripherin-positive and in peripherin-negative DRG neurons, according to some studies, SK3 is a specific immunomarker of SGCs in vivo [32,36]. In addition, the presence of SK3 has been reported in early-cultured cells [36]. To the best of our knowledge, this is the first study that examined and reported an important variation in SK3 staining over time in TGCs in vitro. Our results show that, in the early stages (0–4 h), SK3 is mainly expressed in undifferentiated TG neural cells (Figure 8A). Although a portion of the neurons remained viable during the whole period of evaluation (0–4 h, Figure 11A–C), by 24 h, many neurons were lost, since no specific medium was used for neuron maintenance and growth. Therefore, the SK3 expression significantly decreased (Figure 8 and Figure 9B). After 48 h, undifferentiated TGCs differentiated into SGCs, which better survived and proliferated in the culture medium used. This would explain the rise in the SK3 expression in the immunocytochemistry at 48 h (Figure 8 and Figure 9C). Nonetheless, this hypothesis must be confirmed in future studies. A double staining with SK3 and βIII-tubulin demonstrated, in all stages, cells that were only positive for βIII-tubulin, cells that were only positive for SK3, and cells that showed immunoreactivity for both SK3 and βIII-tubulin. Such findings are presented in the Supplementary Materials (Figure S1A–C). Altogether, these results confirm the great plasticity of SGCs and the important morphological and functional changes that take place in those cells in vitro.

In this study, we used a simplified protocol to examine the potential use of primary TG cultures and the short-term changes that occur in those cells in vitro. Our findings showed that morphological, as well as protein expression, changes started to occur from the earliest hours in murine primary TGC cultures. The results of this study also suggest that early TGC cultures represent an advantage, since they allow for the study of TGCs in a well-controlled environment, which is more similar to what is observed in vivo. Such results confirm and expand on the findings of a previous study [35]. Nonetheless, this is still a controversial issue. A previous study reported the well-preserved morphology of TG cells after 24 h using organ culture [48]. However, that study was conducted in adult rats and not in neonate mice. This could explain the apparently contradictory results.

According to the results of the current article, the developed protocol for primary cultures of TGCs may be a useful platform for use in future pain studies, considering the fewer interventions that may act as confounding factors. The obtained results were compatible with the current literature. However, the combination of several different techniques, such as SEM, a time-lapse, and immunocytochemistry allowed for a better evaluation of how TGCs behave in vitro. Overall, it proved to be a reliable protocol for in vitro studies of sensory ganglia not only the TG, but also the DRG.

This study used neonate mice (P10–P12). This is important when comparing the current findings to the results of other studies [10,49]. In addition, it has been described that, at this this age, neonate mice have a quasi-adult phenotype for some receptors (e.g., P2 × 3 and P2 × 7 receptors) that could potentially be the target of future studies [50].

Despite the absence of the exogenous nerve growth factor (NGF), neurons survived more than 48 h after TG dissociation (Figure 11A–C). This confirms the findings of previous studies [33,35]. The NGF can be considered a confounding factor in pain models, since its release can be found in chronic pain. Therefore, a model that did not supplement the culture medium with NGF was chosen.

What is noteworthy is that this study provided a platform that can be used to investigate the mechanisms of chronic pain, especially trigeminal pain. Nonetheless, no functional evaluation has been tested. This can be considered a limitation of the current study. Future studies that include functional assays will permit the evaluation of the genetic and/or epigenetic regulations, as well as the pathophysiological mechanisms and novel pharmacological approaches applied to treat trigeminal pain using the model presented in this article.

## 5. Conclusions

To date, the physiological and pathological roles of the SGCs, as well as their ionic channels, are not clear. More information is needed regarding this aspect, as well as the glia–neuron interactions in sensory ganglia. This study provides novel information regarding the velocity of the migration of SGCs from neurons in the primary cultures of the TG. In addition, this is the first study that examined and quantified changes in the immunostaining of SK3 throughout the first 48 h of primary cultures of the TG. In addition, SEM and contrast microscopy were able to clearly show differences in the TG cells during 48 h in culture. Notably, this study implemented an easily replicable protocol that might be used in other studies. Nonetheless, further studies are still needed to precisely identify all the factors that control the changes that occur in TG cells in vitro, especially the role of the extracellular matrix elements in this process.

## Data Availability

Data is contained within the article or Supplementary Material.

## Supplementary Materials

The following supporting information can be downloaded at: https://www.mdpi.com/article/10.3390/cimb44030084/s1, Figure S1: Double staining with anti-βIII-tubulin and anti-SK3 performed in cultured TGCs (20× magnification) after 4 h (S1A), 24 h (S1B), and 48 h (S1C). Scale bars represent 100 µm.
