# Supplementary material for: Short-Term Functional and Morphological Changes in the Primary Cultures of Trigeminal Ganglion Cells
Source: Curr Issues Mol Biol. 2022 Mar 8;44(3):1257–72. doi: 10.3390/cimb44030084 (PMC8946888; doi:10.3390/cimb44030084)
Supplement: Supplementary file 1 [file cimb-44-00084-s001.zip › cimb-1629882-supplementary.pdf]

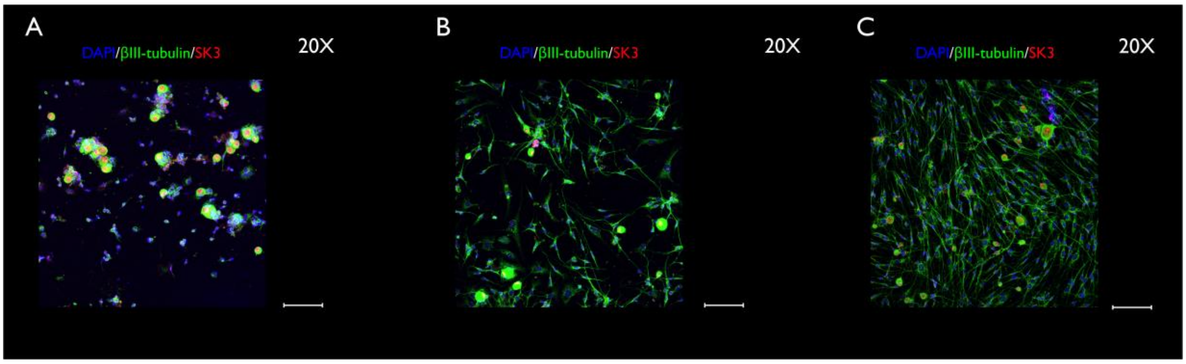

Figure S1. Double staining with anti-βIII-tubulin and anti-SK3 performed in cultured TGCs (20x magnification) after 4h (S1A), 24h (S1B), and 48h (S1C). Scale bars represent 100 μm.
